# Targeted capture enrichment followed by NGS: development and validation of a single comprehensive NIPT for chromosomal aneuploidies, microdeletion syndromes and monogenic diseases

**DOI:** 10.1186/s13039-019-0459-8

**Published:** 2019-11-21

**Authors:** George Koumbaris, Achilleas Achilleos, Michalis Nicolaou, Charalambos Loizides, Kyriakos Tsangaras, Elena Kypri, Petros Mina, Carolina Sismani, Voula Velissariou, Georgia Christopoulou, Pantelis Constantoulakis, Emmanouil Manolakos, Ioannis Papoulidis, Danai Stambouli, Marios Ioannides, Philippos Patsalis

**Affiliations:** 1 NIPD Genetics Public Company Ltd, Neas Engomis 31, Nicosia, 2409 Cyprus; 2 The Cyprus Institute of Neurology and Genetics, International Airport Avenue, 6, Ayios Dometios, Nicosia, 2370 Cyprus; 3 Cyprus School of Molecular Medicine, International Airport Avenue, 6, Ayios Dometios, Nicosia, 2370 Cyprus; 4 Cytogenetics and Molecular Genetics Department, Bioiatriki Healthcare Group, Athens, Greece; 5 Genotypos Science Labs, Athens, Greece; 6 Access To Genome, Genetics Laboratory, Athens-Thessaloniki, Greece; 7 Cytogenomic Medical Laboratory, Bucuresti, Romania

**Keywords:** NIPT, Monogenic diseases, Cell-free DNA, Aneuploidies, Microdeletions

## Abstract

**Background:**

Non-invasive prenatal testing (NIPT) has been widely adopted for the detection of fetal aneuploidies and microdeletion syndromes, nevertheless, limited clinical utilization has been reported for the non-invasive prenatal screening of monogenic diseases. In this study, we present the development and validation of a single comprehensive NIPT for prenatal screening of chromosomal aneuploidies, microdeletions and 50 autosomal recessive disorders associated with severe or moderate clinical phenotype.

**Results:**

We employed a targeted capture enrichment technology powered by custom TArget Capture Sequences (TACS) and multi-engine bioinformatics analysis pipeline to develop and validate a novel NIPT test. This test was validated using 2033 cell-fee DNA (cfDNA) samples from maternal plasma of pregnant women referred for NIPT and paternal genomic DNA. Additionally, 200 amniotic fluid and CVS samples were used for validation purposes. All NIPT samples were correctly classified exhibiting 100% sensitivity (CI 89.7–100%) and 100% specificity (CI 99.8–100%) for chromosomal aneuploidies and microdeletions. Furthermore, 613 targeted causative mutations, of which 87 were unique, corresponding to 21 monogenic diseases, were identified. For the validation of the assay for prenatal diagnosis purposes, all aneuploidies, microdeletions and point mutations were correctly detected in all 200 amniotic fluid and CVS samples.

**Conclusions:**

We present a NIPT for aneuploidies, microdeletions, and monogenic disorders. To our knowledge this is the first time that such a comprehensive NIPT is available for clinical implementation.

## Background

Until recently, prenatal screening for fetal aneuploidies relied on the measurement of maternal serum biochemical markers combined with fetal ultrasound markers. The discovery of cell-free fetal DNA (cffDNA) in maternal circulation prompted the development of non-invasive prenatal testing (NIPT), opening a new era in prenatal screening [1]. Since its introduction, different methods have been applied for the detection of fetal aneuploidies, mainly employing whole genome or targeted approaches combined with Next Generation Sequencing (NGS). The success of such methods has been highlighted in several clinical validation studies that demonstrate the ability for high aneuploidy detection rates [2–9]. This has led to the endorsement of NIPT by several professional bodies as a primary screening method regardless of the pregnancy risk status [10–12]. Furthermore, in the absence of specific markers, NIPT can be used for sex chromosomal aneuploidies and for select microdeletion syndromes [10].

For the non-invasive prenatal screening of monogenic diseases limited clinical utilization has been reported. Early studies were focused on disease detection or exclusion based on the presence of de novo or paternally inherited mutations respectively, using PCR approaches, allowing only for the screening of a limited number of monogenic diseases [13–15]. Recently, the advent of high precision and high throughput technologies such as NGS and digital PCR, catalyzed the development of higher sensitivity assays for the detection of monogenic diseases in cfDNA [16–21], most of them requiring the parental haplotype to interpret the fetal inheritance pattern [18, 22, 23]. Nevertheless, limited scalability, high cost and increased complexity in assay performance and data analysis rendered their clinical implementation challenging [24].

Recently, Luo et al. described in a proof of concept study the feasibility of integrating three tests in a single NIPT for the detection of aneuploidies, large copy number variants (> 20 Mb) and a limited number of single gene diseases using target capture enrichment. The authors employed a direct NIPT approach for screening of monogenic diseases, which is highly dependent on fetal fraction. Despite the promising results, the sensitivity of the assay was low, especially for the detection of point mutations in the cffDNA even in samples with high fetal fraction. As the authors conclude, additional technological optimizations are needed to increase the test’s accuracy and a larger sample cohort is required for validation and determination of the analytical performance of the test prior to clinical implementation [25]. For the determination of the fetal risk for monogenic diseases, unlike Luo et al., we followed a conventional, fetal fraction independent prenatal screening approach by assessing the fetal risk based on the parental carrier status, in the same workflow as cffDNA analysis for aneuploidies and microdeletions.

Addressing the need of NIPT as prenatal screening for both chromosomal and monogenic diseases, we have developed and validated a new, single, comprehensive NIPT providing the fetal risk for these types of genetic conditions. By employing our existing hybrid capture-based technology with minor modifications [7] and multi-engine bioinformatics analysis pipeline, we present the development and validation of a comprehensive NIPT which offers prenatal screening for aneuploidies of chromosomes 13, 18, 21, X, Y, four microdeletion syndromes and 50 autosomal recessive monogenic disorders with severe or moderate clinical phenotype. The fetal risk for aneuploidies and microdeletions is provided based on the analysis of cffDNA present in maternal plasma, whilst the fetal risk for the autosomal recessive monogenic diseases is determined based on Mendelian law of inheritance by combining maternal and paternal carrier status information using cell free maternal DNA (cfmDNA) and paternal DNA respectively.

## Results

The workflow of this novel comprehensive NIPT prenatal screening test for aneuploidies of 13, 18, 21, X, Y, four microdeletion syndromes and 50 single gene diseases, followed by prenatal diagnosis for these disorders in case of high risk pregnancy is described in Additional file 1. The cfDNA, consisting of cffDNA and cfmDNA, from pregnant women of at least 10 weeks of gestation and paternal genomic DNA (*n* = 2033), were simultaneously analyzed for the detection of chromosomal aneuploidies (trisomy 21, trisomy 18 and trisomy 13), sex chromosome aneuploidies (SCA) (monosomy X, Klinefelter syndrome, trisomy X, XYY, and XXYY**),** four microdeletion syndromes (1p36 deletion syndrome, Wolf-Hirschhorn syndrome, Smith-Magenis syndrome and 22q11.2 deletion syndrome) and point mutations for 50 autosomal recessive conditions (Additional file 2).

In the 2033 samples analyzed with this single comprehensive NIPT, we identified all samples correctly, exhibiting 100% sensitivity (CI 89.7–100%) and 100% specificity (CI 99.8–100%) for chromosomal aneuploidies and microdeletion syndromes. Specifically, 22/22 trisomy 21, 4/4 trisomy 18, 1/1 trisomy 13 cases, 2/2 SCAs and 5/5 microdeletion syndromes (three cases of 22q11.2 deletion, one case of Wolf–Hirschhorn syndrome and one case of Smith-Magenis syndrome) were correctly classified (Table 1).

**Table 1:** Assay validation on cfDNA and paternal samples

Description	cfDNA and biological father DNA	Confirmed invasive prenatal diagnosis	Confirmed by Sanger
Total	2033		
Trisomy 21	22	22/22	–
Trisomy 18	4	4/4	–
Trisomy 13	1	1/1	–
SCA	2	2/2	–
Microdeletion syndromes	5	5/5	–
Number of samples with no mutations	1497		496/496^a^
Number of samples with mutations	536		–
Number of mutations	613		–
Number of unique mutations	87		87/87

^**a**^All 496 targeted mutations were confirmed in 5 randomly selected normal (wild-type) samples

The results were in agreement with the invasive prenatal diagnosis results performed by the collaborating laboratories for all tested samples.

**Fig. 1:**
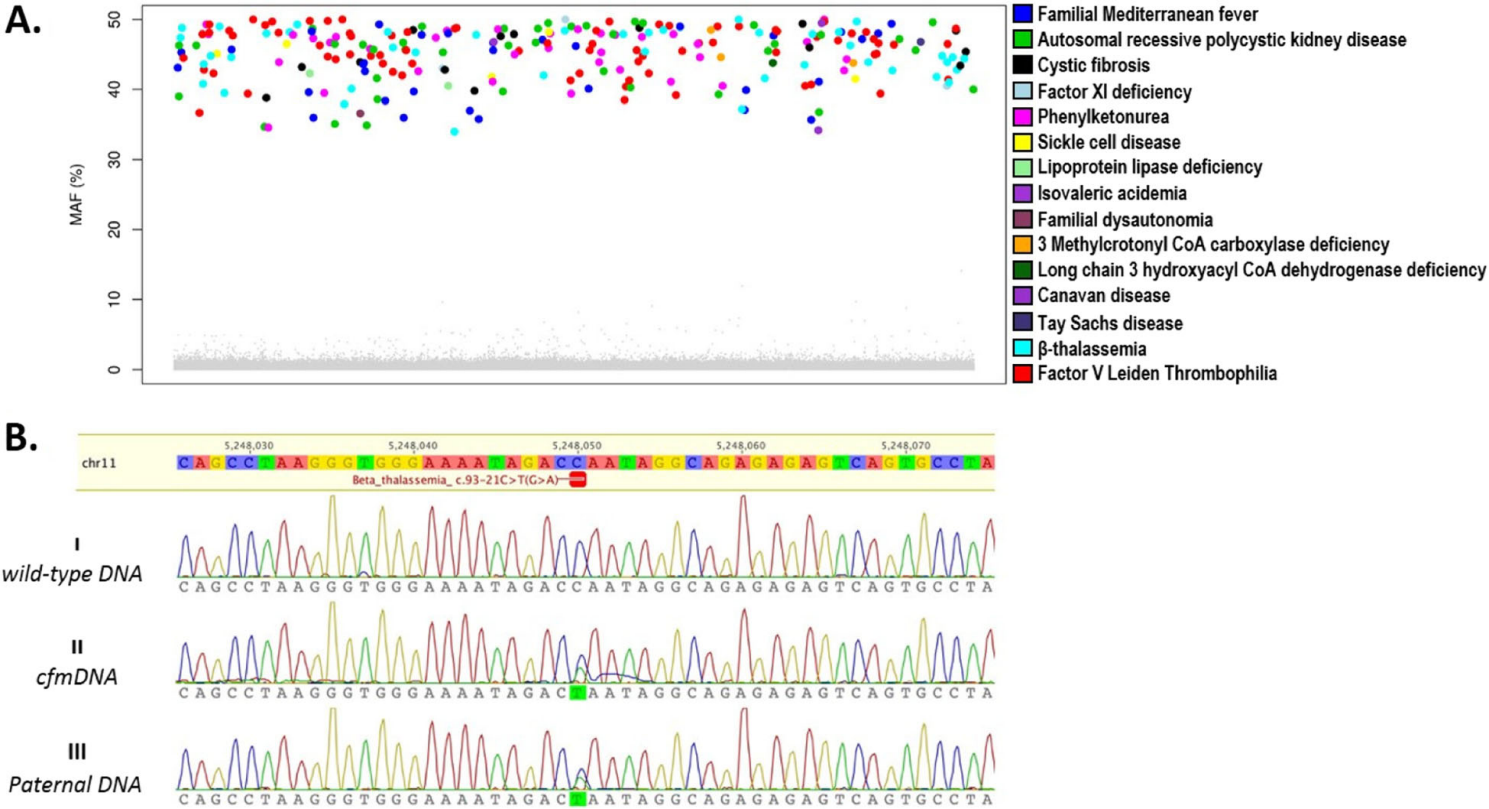
**a** Representative subset of identified point mutations in cfmDNA and paternal DNA. In total 613 (shown 259) targeted causative mutations were detected, corresponding to 21 (shown 15/21) monogenic diseases. Color dots represent the Minor Allele Frequency (MAF) at a targeted mutation as per the legend. Grey dots denote a “negative” call (wild type). **b** Sanger sequencing confirmations of wild-type (I) and parental samples (II and III). A homozygous wild-type sample is shown as normal. Both parents were identified as carriers of a C > T (c.93-21C > T) transition in intron 1 of the HBB gene. In the figure cfmDNA implies cell-free maternal DNA

In the same assay, we identified 1497/2033 samples carrying none of the targeted mutations (wild type) and 536/2033 samples carriers of one or more of the targeted mutations (Table 1). In total, 613 targeted causative mutations were detected, of which 87 were unique (detected at least once), corresponding to 21 monogenic diseases (Table 1). All unique point mutations identified in this cohort were verified by Sanger sequencing using parental genomic DNA (Fig. 1b). All wild-type variants (*n* = 496) were confirmed in five randomly selected normal (wild-type) parental samples (Table 1).

**Fig. 2:**
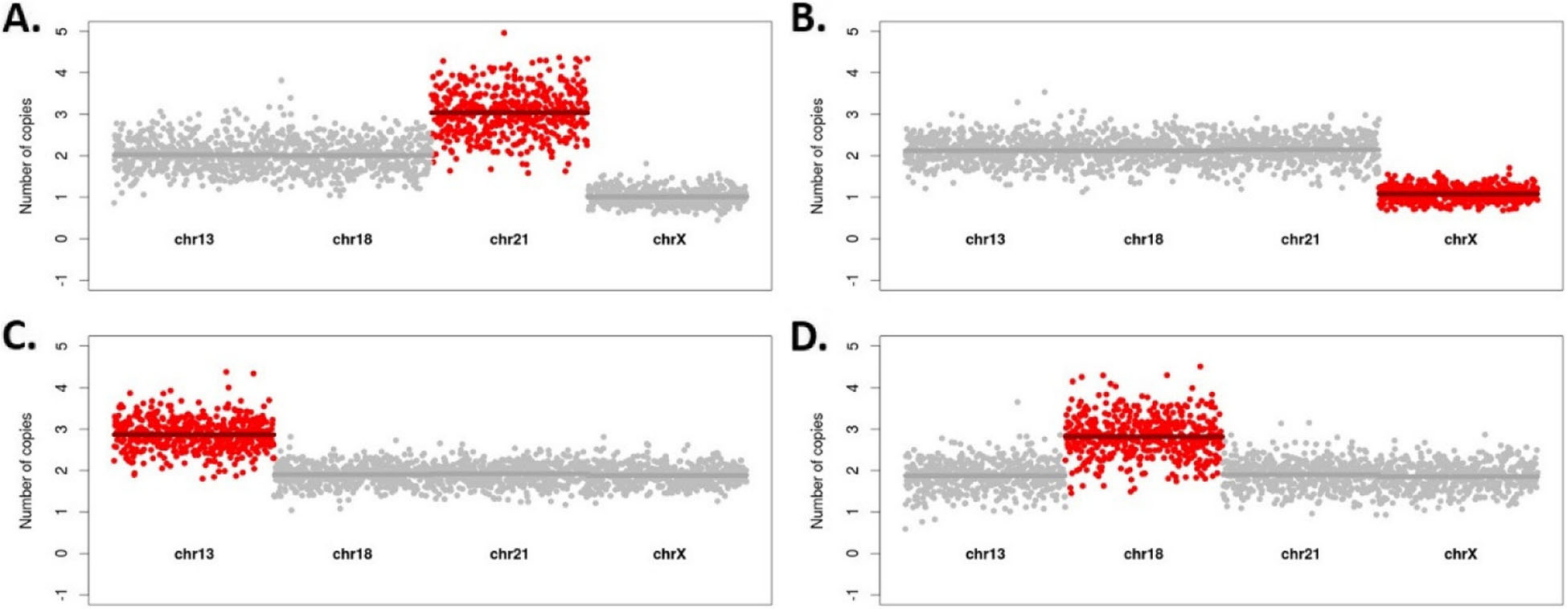
Foetal abnormality detection in prenatal samples. In total, 200 prenatal samples (amniotic fluid and CVS) were subjected to prenatal diagnosis using the same single comprehensive assay. All normal and abnormal samples were correctly classified. The x-axis denotes the targeted chromosomes (chr 13, 18, 21, X). The y-axis represents the normalized read depth per TACS (dots). Red dots denote positive calls. The panels show the detection of **a** Trisomy 21, **b** Monosomy X, **c** Trisomy 13, **d** Trisomy 18

To provide a comprehensive prenatal screening and diagnosis solution, non-invasive results are supplemented with a fetal tissue reflex test using amniotic fluid or CVS for all cases found as high risk for any of the tested conditions (Additional file 1). Therefore, a second retrospective validation study for prenatal diagnosis was performed on 200 amniotic fluid and CVS samples, including 70 aneuploid and six microdeletion samples. Specifically, this targeted test correctly classified all 124 normal, 15 of which were found to carry causative mutations (heterozygous), corresponding to the 50 single gene diseases targeted in our panel (Table 2) and 76 abnormal samples with 100% specificity (CI 97–100%) and 100% sensitivity (CI 95–100%) for aneuploidies and microdeletions (Table 2). The abnormal samples included 41 trisomy 21, nine trisomy 18, six trisomy 13, 14 SCAs, four cases of 22q11.2 deletion and two Wolf–Hirschhorn syndrome cases (Fig. 2).

**Table 2:** Assay validation on amniotic fluid and CVS samples

Description	Normal samples	Abnormal samples
Normal	109/109	–
Aneuploidies of 13, 18, 21, X and Y	–	70/70
Microdeletion syndromes	–	6/6
Single gene diseases	–	15/15^a^

^a^Samples were found to be heterozygous (carriers) for targeted mutations

## Discussion

We have developed and validated a new comprehensive NIPT for fetal aneuploidies of chromosomes 13, 18, 21, X and Y, four microdeletion syndromes (1p36, Wolf-Hirschhorn, Smith-Magenis, 22q11.2) and a panel of 50 autosomal recessive single gene diseases, achieving very high sensitivity and specificity. Furthermore, we validated the assay for prenatal diagnosis using amniotic fluid and CVS, correctly classifying all normal and abnormal samples. As such, the newly developed single comprehensive test provides an extended and validated prenatal screening solution, including prenatal diagnosis for those pregnancies found as high risk for aneuploidies, microdeletions and monogenic diseases.

The benefits of non-invasive prenatal screening for aneuploidies and microdeletions have been thoroughly described, and NIPT is well accepted as an effective, efficient and cost effective prenatal screening for these syndromes [26]. The addition of monogenic diseases to NIPT within a single comprehensive prenatal screening test, as the one presented herein, provides added value to the field of prenatal screening and major benefits for the clinical practice and health care systems.

This single comprehensive NIPT is based on a validated targeted capture enrichment technology [7], offering technological advantages, high accuracy and short turnaround time (TAT). The test can be offered to all pregnant women from the 10th week of gestation onwards, including twin and IVF non-donor pregnancies, allowing timely and informed decisions regarding diagnosis, prevention and better clinical management of the pregnancy. This is the first time that a comprehensive NIPT is made available, setting the first step towards the clinical implementation of NIPT of chromosomal syndromes and monogenic diseases in the field of prenatal screening.

An inherent limitation of NIPT, irrespective of the approach taken, is the discordance between NIPT and prenatal diagnosis mainly due to confined placental mosaicism (CPM). As such, cases with CPM are potential sources of false-positive (FP) and false-negative (FN) results. However, considering the likelihood of the spontaneous abortion of trisomies 13 and 18 by the 12th week of pregnancy and term, and the incidence of trisomy 21 in the general population, the sensitivity and the specificity of NIPT is not considerably affected [7].

In its current form, the test screens for known pathogenic or likely pathogenic mutations covering in most cases higher than 70% detection rate and includes most common mutations reported for each condition (Additional file 4). As such, rare or familial mutations that may be associated with clinical phenotype are not covered by our existing panel. Recent studies have also stressed the importance of prenatal screening of dominant disorders caused by de novo or paternally inherited mutations, as these contribute to nearly 60% of severe postnatal monogenic disease [27]. Acknowledging the clinical relevance and importance of these conditions, work is underway to enrich the existing panel with rare mutations and also include autosomal dominant and X-linked diseases, covering, in this way, all types of monogenic diseases in a single test.

Furthermore, the fetal risk for monogenic diseases is assessed based on the carrier status of the biological parents, therefore, pregnancies achieved using egg donation or surrogate pregnancies are excluded from the fetal risk assessment for monogenic diseases.

Based on the public databases the theoretical risk of being a carrier for at least one of the 50 single gene diseases included in this comprehensive NIPT is 1 in 3, while in our cohort of 2033 samples it was found to be 1 in 4. The actual risk for the fetus being affected by one of the 50 single gene diseases screened by this comprehensive NIPT in our cohort is 1 in 196. The actual total risk of the fetus being affected by one of the autosomal and sex chromosome aneuploidies, four microdeletion syndromes and 50 single gene diseases is 1 in 65 (Additional file 3). Based on the above estimates, all pregnancies matching the characteristics of our cohort should be considered as high risk and screened for these conditions. In the absence of age-associated risk and available biochemical and ultrasound markers for the great majority of monogenic diseases, and given that any pregnancy having such a risk is considered as intermediate to high risk [28, 29], a first tier NIPT such as the one presented herein should be integrated as part of standard health care. Accordingly, being aware of the fetal risk, couples can take informed decisions regarding the clinical management of their pregnancy.

All monogenic diseases in our panel are associated with moderate to severe phenotype or require prenatal or neonatal intervention to improve and manage the pregnancy’s outcome [30]. In accordance to professional bodies’ opinions, we cover only pathogenic or likely pathogenic mutations, with overall disease detection frequency higher than 70% in most cases. Late onset diseases and mutations of unknown significance or mutations not sufficiently documented in the literature were excluded from our panel [31].

A recent population-based study describing couples’ experience with carrier screening revealed that prospective carrier parents are not aware of screening programs, though, upon taking the test, they appreciated knowing their risk thereby recognizing the test’s importance [32]. There are many benefits in combining an extended carrier screening test with a NIPT. Firstly, a single comprehensive prenatal screening test is more efficient, reduces parental anticipation and anxiety, and provides results faster. Secondly, only a minority of couples undergo pre-conception carrier screening, even in countries with the highest level of healthcare provision. Thus, providing a comprehensive solution as the one presented here allows for screening a greater proportion of the potentially affected population. Thirdly, there is higher awareness for NIPT than carrier screening and the uptake of NIPT is constantly increasing, therefore, carrier screening as part of NIPT is more effective. Last but not least, the cost of a comprehensive NIPT compared to carrier screening is significantly lower. In order to maximize the test’s clinical utility, economic implications should also be considered. Even though the cost of a screening test is more easily amortized, compared to the cost of treating an affected child [33, 34], a lower test cost allows for greater uptake. Our assay’s targeted design significantly reduces the number of required reads, resulting in higher efficiency and scalability [7, 35]. By combining all these features we can provide a cost-effective, extended carrier screening and NIPT solution to prospective parents, thereby extending the scope of prenatal care.

## Conclusions

We present, for the first time, the development and validation of a highly accurate single and comprehensive NIPT for aneuploidies, microdeletions and a high number of monogenic diseases opening a new chapter in the field of Non-Invasive Prenatal Screening. The test presented here is based on a target capture enrichment and utilizes specific TACS designed to interrogate selected regions on chromosomes 13, 18, 21, X, Y, critical regions of 22q11.2, 1p36, Wolf-Hirschhorn, Smith-Magenis microdeletion syndromes and locations of known causative mutations involved in 50 autosomal recessive disorders, including life threatening conditions, moderate or severe impairments, congenital anomalies, developmental delay, hearing loss, blindness and metabolic disorders. By leveraging the inherent high enrichment uniformity and read depth of the assay in combination with multi-engine bioinformatics, we achieved higher than 99% accuracy for the tested diseases, offering the same technological advantages, fast turnaround time, unrivalled classification and accurate fetal fraction estimation as previously described [7].

## Methods

### Sample collection

Protocols used for sample collection were approved by the Cyprus National Bioethics Committee (EEBK/EΠ/2011/14) and informed consent was obtained from all participants. The study was performed in accordance with the ethical standards of the institutional and/or national research committee and with the 1964 Helsinki Declaration.

For the retrospective validation of the comprehensive NIPT, a total of 2033 samples of known status (normal or abnormal for aneuploidy and microdeletion syndromes) were used which included cfDNA samples isolated from pregnant women with moderate to high risk pregnancies and genomic DNA isolated from the biological fathers. cfDNA was isolated from maternal peripheral blood samples (8 ml) collected from pregnant women of at least 18 years of age, referred for NIPT from the 10th week of gestation onwards, in BCT StreckTubes (Streck Inc., Omaha, NE). A mean of 4 ml plasma was isolated from all maternal peripheral blood samples via a double centrifugation protocol as previously described [7]. Genomic DNA from the biological father was isolated from self-collected buccal swab samples in ‘hDNA free’ FLOQSwabs (COPAN, Italy) following provided instructions.

For the retrospective prenatal diagnosis validation study, 200 previously characterized amniotic and CVS samples were sent to NIPD Genetics by collaborating laboratories.

### DNA isolation

Circulating cfDNA was extracted from 4 ml plasma using the QIAsymphony SP and the Qiasymphony Circulating DNA kit (Qiagen, Hilden, Germany) following manufacturer’s instructions. Genomic DNA was isolated from buccal swabs, amniotic fluid or CVS samples using the QIAamp Mini kit (Qiagen) following manufacturer’s instructions.

### Validation

For the single comprehensive NIPT validation study, the status of all 2033 samples was known. All abnormal samples were previously confirmed by QF-PCR and/or complete chromosomal analysis by Chromosomal Microarray Analysis (CMA) or conventional karyotyping by the collaborating centers, after invasive procedures. At the time of testing the parental carrier status of all 2033 samples was unknown to those handling the samples (Table 1). Thus, the identification of normal constitution or detection of targeted mutations using the single comprehensive NIPT was confirmed on parental genomic DNA using Sanger sequencing. Prior to the study the samples identity was coded to allow for a blind validation study.

This comprehensive NIPT includes prenatal diagnosis confirmation for all high-risk NIPT samples (Additional file 1). Towards this goal, a retrospective validation study was performed using fetal tissues, i.e. amniotic fluid or chorionic villus sampling (CVS). In total, an independent set of 200 amniotic fluid or CVS samples were tested (Table 2). All 200 samples had been previously analyzed using standard prenatal diagnosis methodologies, including cytogenetic analysis and CMA. The sample identity was coded to allow for a blind validation study.

### Selection of 50 single-gene diseases for prenatal screening

The 50 monogenic autosomal recessive diseases included in the NIPT panel were selected to provide clinical utility and prognostic value in prenatal screening (Additional file 2). They are all associated with a moderate to severe clinical phenotype. These include life-threatening conditions or conditions leading to moderate or severe impairments, congenital anomalies, developmental delay, hearing loss, blindness, metabolic disorders, etc. Although in several cases these conditions may be rare in the general (world-wide) population, they are encountered frequently in the high risk populations.

Only known pathogenic, or likely pathogenic variants were included during the selection of causative mutations (Additional file 4). In cases where a database dedicated specifically to the condition of interest was available, for example the cystic fibrosis database (The Clinical and Functional TRanslation of CFTR (CFTR2); available at http://cftr2.org; true on 27/11/2018), it was consulted in conjunction with references therein to obtain or deduce the detection frequencies for individual mutations in specific populations. Where no databases were available, a thorough review of the literature was performed for the selection of variants which have a clear correlation with the disease in specific populations (e.g. Tay Sachs, abetalipoproteinemia and Canavan disease in Ashkenazi Jews).

In total, 496 causative mutations are targeted, located on 49 genes with cumulative disease detection frequency higher than 70% (Additional file 4). For conditions where a very large number of mutations has been reported and most of the mutations tend to be rare and private/familial, such as 3-methylcrotonyl-CoA carboxylase deficiency, the most common pathogenic variants were selected, providing the highest detection frequency in the corresponding populations.

### Sequencing library preparation

The cfDNA was subjected to library preparation as previously described [7] with modifications. In brief, initially blunt ending and 5′ phosphorylation was performed using T4 polymerase and T4 kinase respectively. Sequencing adaptors were then ligated at both ends using T4 Ligase (New England Biolabs, Ipswich, UK). Nicks were removed in a fill-in reaction using Bst polymerase (New England Biolabs). Library amplification was performed using Herculase II Fusion Polymerase (Agilent Technologies, Santa Clara, CA), and unique barcodes were assigned to all samples. At each step, products were purified using Ampure XP magnetic beads according to manufacturer’s instructions.

Prior to library construction of buccal swab, amniotic fluid and CVS samples, extracted genomic DNA was sheared to an average size of 250 bp using the Bioruptor Pico sonication system (Diagenode, Liege, Belgium). Blunt ending, adaptor ligation and adaptor fill-in reactions were performed without intermediate purification steps. A single purification step was performed following library amplification using Ampure XP magnetic beads according to manufacturer’s instructions.

All library preparation steps were run on Hamilton STAR (Hamilton, Bonaduz, Switzerland) or epMotion (Eppendorf) systems using in-house developed automated methods.

### Design and preparation of target-capture sequences

TArget Capture Sequences (TACS) were used to enrich regions of interest on chromosomes 13, 18, 21, X, Y and critical regions of 22q11.2, 1p36, Wolf-Hirschhorn and Smith-Magenis microdeletion syndromes. TACS were also specifically designed based on genomic locations of known causative mutations involved in 50 autosomal recessive disorders. Target loci were selected based on GC content, distance from repetitive elements and absence of known surrounding complex architecture [7].

For the preparation of TACS, PCR was performed using MyTaq polymerase (Bioline, London, UK) using normal DNA followed by purification using Ampure XP magnetic beads. TACS were quantified using the NanoDrop-ND8000 (Thermo Scientific, Wilmington, DE, USA) and were pooled equimolarly. The final mix was blunt-ended using the Quick Blunting kit (New England Biolabs) and biotinylated using the Quick Ligation kit (New England Biolabs). Products were purified using Ampure XP magnetic beads.

### In-solution hybridization and sequencing

Each amplified library was mixed with 2 × hybridization buffer (Agilent Technologies), 10× blocking agent (Agilent Technologies), blocking oligonucleotides, Cot-1 DNA (Invitrogen, Carlsbad, CA), and salmon sperm DNA (Invitrogen). The hybridization reaction mix was denatured at 95 °C for 3 min followed by blocking incubation at 37 °C before being added to the biotinylated TACS. The samples were then incubated at 65 °C for 16 h. After hybridization, unbound DNA was washed and captured sequences were eluted by heating as previously described [7]. All capture steps were performed on a Hamilton STARlet liquid handler using in-house developed methods.

Enriched samples were then amplified using outerbound primers, pooled equimolarly and sequenced using a NextSeq 500 sequencer (illumina).

### Data analysis

The sequencing data of the enriched samples was analyzed using methods as described in Koumbaris et al. with modifications [7]. The cfDNA and paternal genomic DNA samples were further processed to compute the parental carrier status and subsequently determine the fetal risk for the targeted monogenic diseases. For parental carrier status determination, the Variant Allele Frequency (VAF) at each targeted locus was used. Specifically, we used the number of times the predefined variant allele was sequenced over the total number of times the locus was sequenced (Additional file 5).

For the paternal sample (buccal swab), the expected VAF for heterozygous loci (carrier) and homozygous loci is 0.5 and 1 respectively. In maternal samples, the expected value of the VAF is dependent on the cell free maternal fraction present in the plasma. Hence, a carrier mother is expected to have (i) a VAF value equal to half the maternal fraction if the fetus has only the wild type allele and (ii) a VAF value greater than half the maternal fraction if the fetus is either heterozygous or homozygous for the variant allele. The algorithm estimates the carrier status of each of the parents using proportions tests (Additional file 5).

The fetal risk for inheriting the targeted genetic condition was determined from the estimated carrier status of the parents following the Mendelian law of inheritance. Specifically, if both parents are carriers for the same recessive genetic condition, then the fetus has a 25% chance of inheriting the genetic condition. In such cases the pregnancy would be considered as “high risk” for the monogenic disease. All amniotic fluid and CVS samples were processed using the bioinformatics pipeline previously described [7]. Copy number estimation for all targeted regions is achieved using data normalization and change-point detection methods [7, 36] .

## Supplementary information

**Additional file 1: Figure S1.** Workflow of the new single comprehensive NIPT for aneuploidies, microdeletions and single gene diseases. (Left side) Cell-free fetal DNA (cffDNA) is analyzed for fetal risk determination for chromosomal aneuploidies (trisomy 21, trisomy 18 and trisomy 13), sex chromosome aneuploidies (SCA) and four microdeletion syndromes using a custom multi-engine bioinformatics analysis pipeline. In the same assay, cell-free maternal (cfmDNA) and genomic DNA from the biological father are subjected in-solution hybridization enrichment for parental carrier status determination for 50 autosomal recessive conditions. The fetal risk for inheriting a genetic condition is determined following the Mendelian law of inheritance. A pregnancy is considered as “high risk” if both parents are carriers of the same autosomal recessive disease. (Right side) Following a high risk NIPT result for an aneuploidy or microdeletion or single gene diseases prenatal diagnosis is offered by analysis of amniotic fluid or CVS.

**Additional file 2: Table S1.** List of 50 monogenic disorders included in the targeted disease panel.

**Additional file 3: Table S2.** The theoretical and estimated risk for the parents and the fetus.

**Additional file 4: Table S3**. List of 50 monogenic diseases screened by comprehensive NIPT. Targeted mutations covered are outlined along with detection frequencies in corresponding populations

**Additional file 5: Figure S2.** Flowchart illustrating the bioinformatics analysis pipeline for a typical sequencing run consisting of plasma samples. The same pipeline applies for paternal samples with the last step being performed for variant calling (monogenic diseases) only.

## Data Availability

All supporting data are included in the manuscript.

## Abbreviations

cfDNA: Cell-free DNA
cffDNA: Cell-free fetal DNA
cfmDNA: Cell-free maternal DNA
CPM: Confined Placental Mosaicism
FN: False Negative
FP: False Positive
NGS: Next Generation Sequencing
NIPT: Non-invasive prenatal testing
SCA: Sex chromosome aneuploidies
TACS: Target Capture sequences
