# Supplementary material for: Targeted capture enrichment followed by NGS: development and validation of a single comprehensive NIPT for chromosomal aneuploidies, microdeletion syndromes and monogenic diseases
Source: Mol Cytogenet. 2019 Nov 21;12:48. doi: 10.1186/s13039-019-0459-8 (PMC6873497; doi:10.1186/s13039-019-0459-8)
Supplement: Supplementary file 1 — Additional file 1: Figure S1. Workflow of the new single comprehensive NIPT for aneuploidies, microdeletions and single gene diseases. (Left side) Cell-free fetal DNA (cffDNA) is analyzed for fetal risk determination for chromosomal aneuploidies (trisomy 21, trisomy 18 and trisomy 13), sex chromosome aneuploidies (SCA) and four microdeletion syndromes using a custom multi-engine bioinformatics analysis pipeline. In the same assay, cell-free maternal (cfmDNA) and genomic DNA from the biological father are subjected in-solution hybridization enrichment for parental carrier status determination for 50 autosomal recessive conditions. The fetal risk for inheriting a genetic condition is determined following the Mendelian law of inheritance. A pregnancy is considered as “high risk” if both parents are carriers of the same autosomal recessive disease. (Right side) Following a high risk NIPT result for an aneuploidy or microdeletion or single gene diseases prenatal diagnosis is offered by analysis of amniotic fluid or CVS. [file 13039_2019_459_MOESM1_ESM.docx]

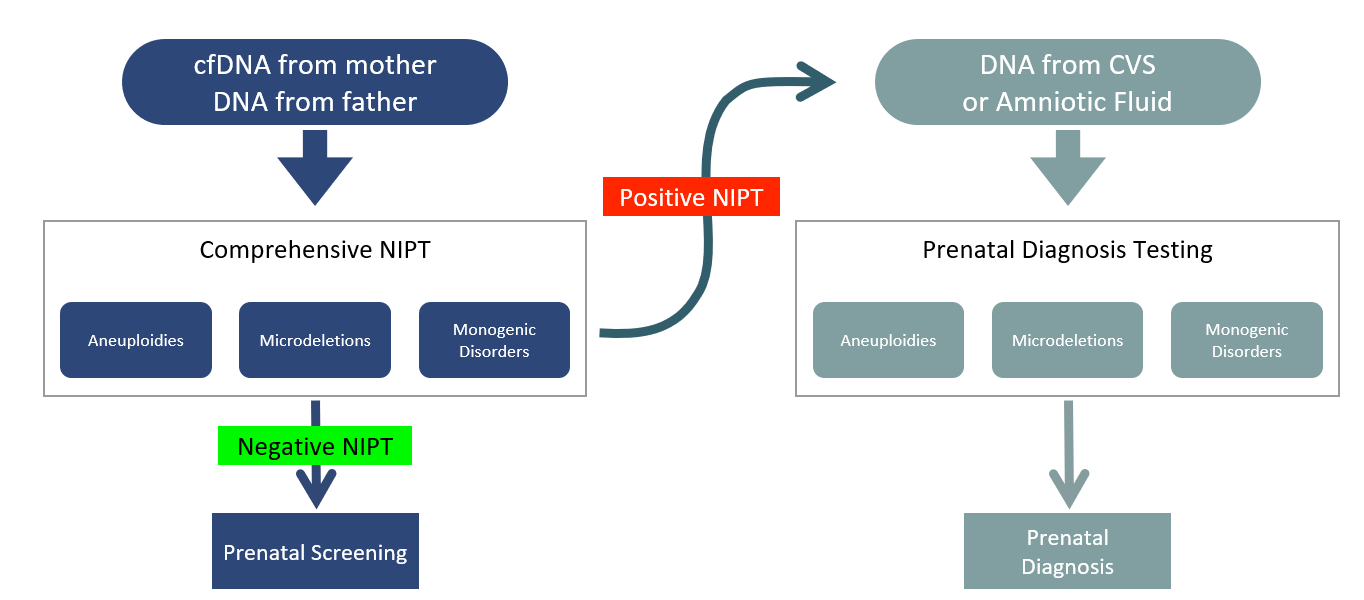


Workflow of the new single comprehensive NIPT for aneuploidies, microdeletions and single gene diseases. (Left side) Cell-free fetal DNA (cffDNA) is analyzed for fetal risk determination for chromosomal aneuploidies (trisomy 21, trisomy 18 and trisomy 13), sex chromosome aneuploidies (SCA) and four microdeletion syndromes using a custom multi-engine bioinformatics analysis pipeline. In the same assay, cell-free maternal (cfmDNA) and genomic DNA from the biological father are subjected in-solution hybridization enrichment for parental carrier status determination for 50 autosomal recessive conditions. The fetal risk for inheriting a genetic condition is determined following the Mendelian law of inheritance. A pregnancy is considered as “high risk” if both parents are carriers of the same autosomal recessive disease. (Right side) Following a high risk NIPT result for an aneuploidy or microdeletion or single gene diseases prenatal diagnosis is offered by analysis of amniotic fluid or CVS.
