# Supplementary material for: Targeted capture enrichment followed by NGS: development and validation of a single comprehensive NIPT for chromosomal aneuploidies, microdeletion syndromes and monogenic diseases
Source: Mol Cytogenet. 2019 Nov 21;12:48. doi: 10.1186/s13039-019-0459-8 (PMC6873497; doi:10.1186/s13039-019-0459-8)
Supplement: Supplementary file 2 — Additional file 2: Table S1. List of 50 monogenic disorders included in the targeted disease panel. [file 13039_2019_459_MOESM2_ESM.docx]

| **Condition** | **Gene** | **OMIM** | **Phenotype MIM** |
| --- | --- | --- | --- |
| Abetalipoproteinemia | *MTTP* | 157147 | 200100 |
| Arthrogryposis, mental retardation seizures | *SLC35A3* | 605632 | 615553 |
| Autosomal recessive polycystic kidney disease | *PKHD1* | 606702 | 263200 |
| Bardet Biedl syndrome 12 | *BBS12* | 610683 | 615989 |
| Beta thalassemia | *HBB* | 141900 | 613985 |
| Canavan disease | *ASPA* | 608034 | 271900 |
| Choreacanthocytosis | *VPS13A* | 605978 | 200150 |
| Crigler Najjar syndrome, Type I | *UGT1A1* | 191740 | 218800 |
| Cystic fibrosis | *CFTR* | 602421 | 219700 |
| Factor V Leiden thrombophilia | *F5* | 612309 | 227400 |
| Factor XI deficiency | *F11* | 264900 | 612416 |
| Familial dysautonomia | *IKBKAP* | 603722 | 223900 |
| Familial Mediterranean fever | *MEFV* | 608107 | 249100 |
| Fanconi anemia (FANCG-related) | *FANCG* | 602956 | 614082 |
| Glycine encephalopathy (GLDC-related) | *GLDC* | 238300 | 605899 |
| Glycogen storage disease, Type 3 | *AGL* | 610860 | 232400 |
| Glycogen storage disease, Type 7 | *PFKM* | 610681 | 232800 |
| GRACILE syndrome | *BCS1L* | 603647 | 603358 |
| Inclusion body myopathy, type 2 | *GNE* | 603824 | 605820 |
| Isovaleric acidemia | *IVD* | 607036 | 243500 |
| Joubert syndrome, type 2 | *TMEM216* | 613277 | 608091 |
| Junctional epidermolysis bullosa, Herlitz type | *LAMC2* | 150292 | 226700 |
| Leber congenital amaurosis (LCA5-related) | *LCA5* | 611408 | 604537 |
| Leydig cell hypoplasia [Luteinizing hormone resistance] | *LHCGR* | 152790 | 238320 |
| Limb girdle muscular dystrophy, type 2E | *SGCB* | 600900 | 604286 |
| Lipoamide dehydrogenase deficiency [Maple syrup urine disease, type 3] | *DLD* | 238331 | 246900 |
| Lipoprotein lipase deficiency | *LPL* | 609708 | 238600 |
| Long chain 3-hydroxyacyl-CoA dehydrogenase deficiency | *HADHA* | 600890 | 609016 |
| Maple syrup urine disease, type 1B | *BCKDHB* | 248611 | 248600 |
| Methylmalonic acidemia (MMAA-related) | *MMAA* | 607481 | 251100 |
| Multiple sulfatase deficiency | *SUMF1* | 607939 | 272200 |
| Navajo neurohepatopathy [MPV17-related hepatocerebral mitochondrial DNA depletion syndrome] | *MPV17* | 137960 | 256810 |
| Neuronal ceroid lipofuscinosis (MFSD8-related) | *MFSD8* | 611124 | 610951 |
| Nijmegen breakage syndrome | *NBN* | 602667 | 251260 |
| Ornithine translocase deficiency [Hyperornithinemia-Hyperammonemia-Homocitrullinuria (HHH) Syndrome] | *SLC25A15* | 603861 | 238970 |
| Peroxisome biogenesis disorders, Zellweger syndrome spectrum (PEX1-related) | *PEX1* | 602136 | 214100 |
| Peroxisome biogenesis disorders, Zellweger syndrome spectrum (PEX2-related) | *PEX2* | 170993 | 614866 |
| Phenylketonurea | *PAH* | 612349 | 261600 |
| Pontocerebellar hypoplasia, type 2E | *VPS53* | 615850 | 615851 |
| Pycnodysostosis | *CTSK* | 601105 | 265800 |
| Pyruvate dehydrogenase deficiency (PDHB-related) | *PDHB* | 179060 | 614111 |
| Retinal dystrophy (RLBP1-related) [Bothnia retinal dystrophy] | *RLBP1* | 180090 | 607475 |
| Retinitis pigmentosa (DHDDS-related) | *DHDDS* | 608172 | 613861 |
| Sanfilippo syndrome, type D [Mucopolysaccharidosis IIID] | *GNS* | 607664 | 252940 |
| Sickle-cell disease | *HBB* | 141900 | 603903 |
| Sjögren-Larsson syndrome | *ALDH3A2* | 609523 | 270200 |
| Tay-Sachs disease | *HEXA* | 606869 | 272800 |
| Usher syndrome, type 1F | *PCDH15* | 605514 | 602083 |
| 3 methylcrotonyl CoA carboxylase deficiency 1 | *MCCC1* | 609010 | 210200 |
| 3 methylcrotonyl CoA carboxylase deficiency 2 | *MCCC2* | 609014 | 210210 |

List of 50 monogenic disorders included in the targeted disease panel.
