# Supplementary material for: Targeted capture enrichment followed by NGS: development and validation of a single comprehensive NIPT for chromosomal aneuploidies, microdeletion syndromes and monogenic diseases
Source: Mol Cytogenet. 2019 Nov 21;12:48. doi: 10.1186/s13039-019-0459-8 (PMC6873497; doi:10.1186/s13039-019-0459-8)
Supplement: Supplementary file 3 — Additional file 3: Table S2. The theoretical and estimated risk for the parents and the fetus. [file 13039_2019_459_MOESM3_ESM.docx]

The theoretical and estimated risk for the parents and the fetus.

| **Risk for the pregnancy and the fetus** | **Risk** |
| --- | --- |
| Theoretical risk of being a carrier for at least one of the 50 single gene diseases screened by the comprehensive NIPT^(a)^ | 1/3 |
| Empirical risk of being a carrier for at least one of the 50 single gene diseases screened by the comprehensive NIPT in our cohort | 1/4 |
| Empirical risk for the fetus to be affected for one of the 50 single gene diseases screened by the comprehensive NIPT in our cohort | 1/196 |
| Empirical risk for the fetus to be affected for one of the aneuploidies, four microdeletion syndromes and 50 single gene diseases screened by the comprehensive NIPT in our cohort^(b)^ | 1/65 |

The theoretical and empirical risk for the parents and the fetus. (a) Carrier frequency is based on numbers obtained from online databases (Orphanet, GeneReviews®, The Human Gene Mutation Database [HGMD], Online Mendelian Inheritance in Man® [OMIM]) and references therein. Rates of affected pregnancies were calculated for each condition using the carrier rates. We consider that an affected fetus is the result of two carrier parents with 25% probability; i.e.: affected rate = (carrier rate)^2^x25%. The cumulative affected rate is taken to be the sum of all the individual condition rates. (b) 1/100 for 13,18,21,X,Y,MDs + 1/196 for the single gene disease in the general population.
