# Supplementary material for: Targeted capture enrichment followed by NGS: development and validation of a single comprehensive NIPT for chromosomal aneuploidies, microdeletion syndromes and monogenic diseases
Source: Mol Cytogenet. 2019 Nov 21;12:48. doi: 10.1186/s13039-019-0459-8 (PMC6873497; doi:10.1186/s13039-019-0459-8)
Supplement: Supplementary file 5 — Additional file 5: Figure S2. Flowchart illustrating the bioinformatics analysis pipeline for a typical sequencing run consisting of plasma samples. The same pipeline applies for paternal samples with the last step being performed for variant calling (monogenic diseases) only. [file 13039_2019_459_MOESM5_ESM.docx]

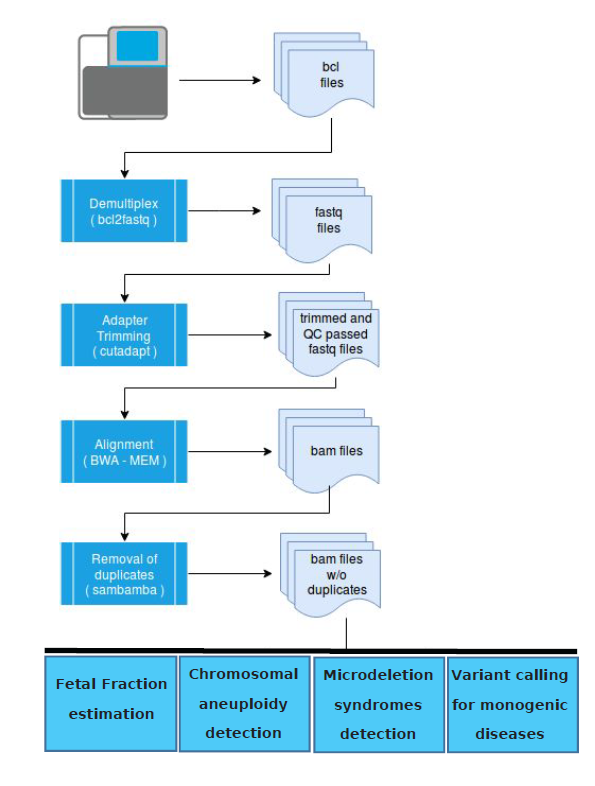


Flowchart illustrating the bioinformatics analysis pipeline for a typical sequencing run consisting of plasma samples. The same pipeline applies for paternal samples with the last step being performed for variant calling (monogenic diseases) only.
